# Comparative acetylomic analysis reveals differentially acetylated proteins regulating fungal metabolism in hypovirus‐infected chestnut blight fungus

**DOI:** 10.1111/mpp.13358

**Published:** 2023-06-06

**Authors:** Ru Li, Fengyue Chen, Shuangcai Li, Luying Yuan, Lijiu Zhao, Shigen Tian, Baoshan Chen

**Affiliations:** ^1^ State Key Laboratory for Conservation and Utilization of Subtropical Agro‐Bioresources, College of Life Science and Technology Guangxi University Nanning China; ^2^ Guangxi Key Laboratory of Sugarcane Biology, College of Agriculture Guangxi University Nanning China

**Keywords:** acetylome, *Cryphonectria parasitica*, hypovirus, metabolism, virulence

## Abstract

*Cryphonectria parasitica*, the chestnut blight fungus, and hypoviruses are excellent models for examining fungal pathogenesis and virus–host interactions. Increasing evidence suggests that lysine acetylation plays a regulatory role in cell processes and signalling. To understand protein regulation in *C*. *parasitica* by hypoviruses at the level of posttranslational modification, a label‐free comparative acetylome analysis was performed in the fungus with or without Cryphonectria hypovirus 1 (CHV1) infection. Using enrichment of acetyl‐peptides with a specific anti‐acetyl‐lysine antibody, followed by high accuracy liquid chromatography–tandem mass spectrometry analysis, 638 lysine acetylation sites were identified on 616 peptides, corresponding to 325 unique proteins. Further analysis revealed that 80 of 325 proteins were differentially acetylated between *C. parasitica* strain EP155 and EP155/CHV1‐EP713, with 43 and 37 characterized as up‐ and down‐regulated, respectively. Moreover, 75 and 65 distinct acetylated proteins were found in EP155 and EP155/CHV1‐EP713, respectively. Bioinformatics analysis revealed that the differentially acetylated proteins were involved in various biological processes and were particularly enriched in metabolic processes. Differences in acetylation in *C*. *parasitica* citrate synthase, a key enzyme in the tricarboxylic acid cycle, were further validated by immunoprecipitation and western blotting. Site‐specific mutagenesis and biochemical studies demonstrated that the acetylation of lysine‐55 plays a vital role in the regulation of the enzymatic activity of *C*. *parasitica* citrate synthase in vitro and in vivo. These findings provide a valuable resource for the functional analysis of lysine acetylation in *C*. *parasitica*, as well as improving our understanding of fungal protein regulation by hypoviruses from a protein acetylation perspective.

## Graphical abstract

Comparison of *Cryphonectria parasitica* with or without hypovirus found differentially acetylated proteins were enriched in metabolic processes; the acetylation of citrate synthase lysine‐55 plays a vital role in its activity.

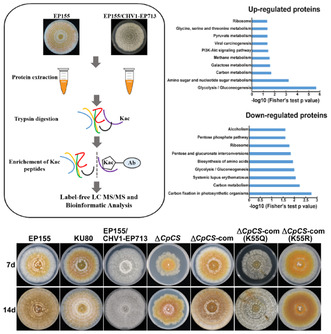

## 1 INTRODUCTION

Protein acetylation is a dynamic and highly conserved posttranslational modification in prokaryotes and eukaryotes (Hart & Ball, 2013). Histone acetylation was first found to be closely related to transcriptional regulation (Allfrey et al., 1964). Acetylation is also found in many nonhistone proteins such as transcription factors, nuclear‐related proteins, hormone receptors, cell metabolism‐related proteins, and cancer‐related proteins (Batta et al., 2007; Spange et al., 2009). Through the reversible addition of an acetyl group to lysine residues, protein acetylation regulates enzymatic activity, protein localization, protein stability, and protein–protein and protein–nucleic acid interactions (Arif et al., 2010). Recent advances in high‐affinity purification of lysine‐acetylated peptides and high‐resolution mass spectrometry have promoted the development of the lysine acetylome in many eukaryotes and prokaryotes. Proteome‐wide analyses have led to the discovery of various cellular functions of lysine acetylation, especially those associated with central metabolic pathways (Henriksen et al., 2012; Nie et al., 2015; Weinert et al., 2011; Wu et al., 2011; Xie et al., 2015; Zhang et al., 2013).

Recent evidence suggests that lysine acetylation is common in fungi. In *Candida albicans*, 477 of 9038 (5.28%) proteins were found to be acetylated. This first study of acetylated proteins in human‐pathogenic fungi provides an important basis for further studies on the functional analysis of acetylated proteins in pathogenic fungi (Zhou et al., 2016). Further knowledge of lysine acetylation in human‐pathogenic fungi was obtained from *Histoplasma capsulatum* (Xie et al., 2016), *Trichophyton rubrum* (Xu et al., 2018), *Cryptococcus neoformans* (Brandao et al., 2018), and *Aspergillus fumigatus* (Lin et al., 2020). In plant‐pathogenic fungi, systematic analyses of the lysine acetylome have been performed in *Fusarium graminearum* (Zhou & Wu, 2019), *Phytophthora sojae* (Li, Lv, et al., 2016), *Botrytis cinerea* (Lv et al., 2016), *Magnaporthe oryzae* (Liang et al., 2018), and *Aspergillus flavus* (Yang et al., 2019). These reports demonstrate that lysine acetylation not only regulates important cellular processes, such as enzyme activity, signal transduction, cell division, and metabolism, but also morphological transition, stress response, biofilm formation, and other processes, thus regulating the fungal life cycle (Cheng et al., 2016; Kim et al., 2015; Narita et al., 2019). However, the relationship between the specific mechanism of acetylation and fungal virulence regulation is yet to be determined.

The ascomycete fungus *Cryphonectria parasitica* is the causal agent of chestnut blight disease, which destroys billions of American chestnut trees (Rigling & Prospero, 2018). The fungus hosts a wide range of viruses and serves as a useful model to examine virus–host interactions and fungal pathogenesis (Eusebio‐Cope et al., 2015). Wild‐type *C*. *parasitica* strain EP155 is an orange‐pigmented, virulent, hypovirus‐free strain that can induce large cankers on chestnut stems. Fungal virulence is attenuated when infected with hypoviruses, a group of single‐stranded, positive‐sense RNA viruses (Dawe & Nuss, 2001). Furthermore, hypovirus infection alters the phenotypic traits of fungi, such as reduced growth rate and pigmentation, loss of asexual sporulation, and suppression of female sterility (Nuss, 2005). Many efforts have been made to understand the mechanism of hypovirulence and comparative transcriptomic, proteomic, metabolomic, and methylomic research has revealed a wide range of hypovirus‐regulated and virulence‐related proteins (Allen et al., 2003; Chun et al., 2020; Dawe et al., 2009; Li et al., 2018; Shang et al., 2008; Wang et al., 2014). Nevertheless, the detailed regulatory mechanisms of protein expression, particularly at the posttranslational level, are unexplored.

In this study, the impact of hypoviral infection on lysine acetylation in *C*. *parasitica* was investigated by generating and comparing two fungal strain acetylomes using label‐free quantitative proteomics. The different acetylation levels of *C*. *parasitica* citrate synthase (CpCS), a key enzyme in the tricarboxylic acid (TCA) cycle, were further verified by immunoprecipitation and western blotting. The functional significance of the lysine acetylation (Kac) site on CpCS was confirmed by site‐specific mutagenesis and biochemical studies. Our study provides extensive data about lysine acetylation in *C*. *parasitica* for the first time and reveals novel insights into the relationship between hypovirulence and protein acetylation.

## 2 RESULTS

### 2.1 Hypovirus infection affects the global acetylome of *C*. *parasitica*

**FIGURE 1:**
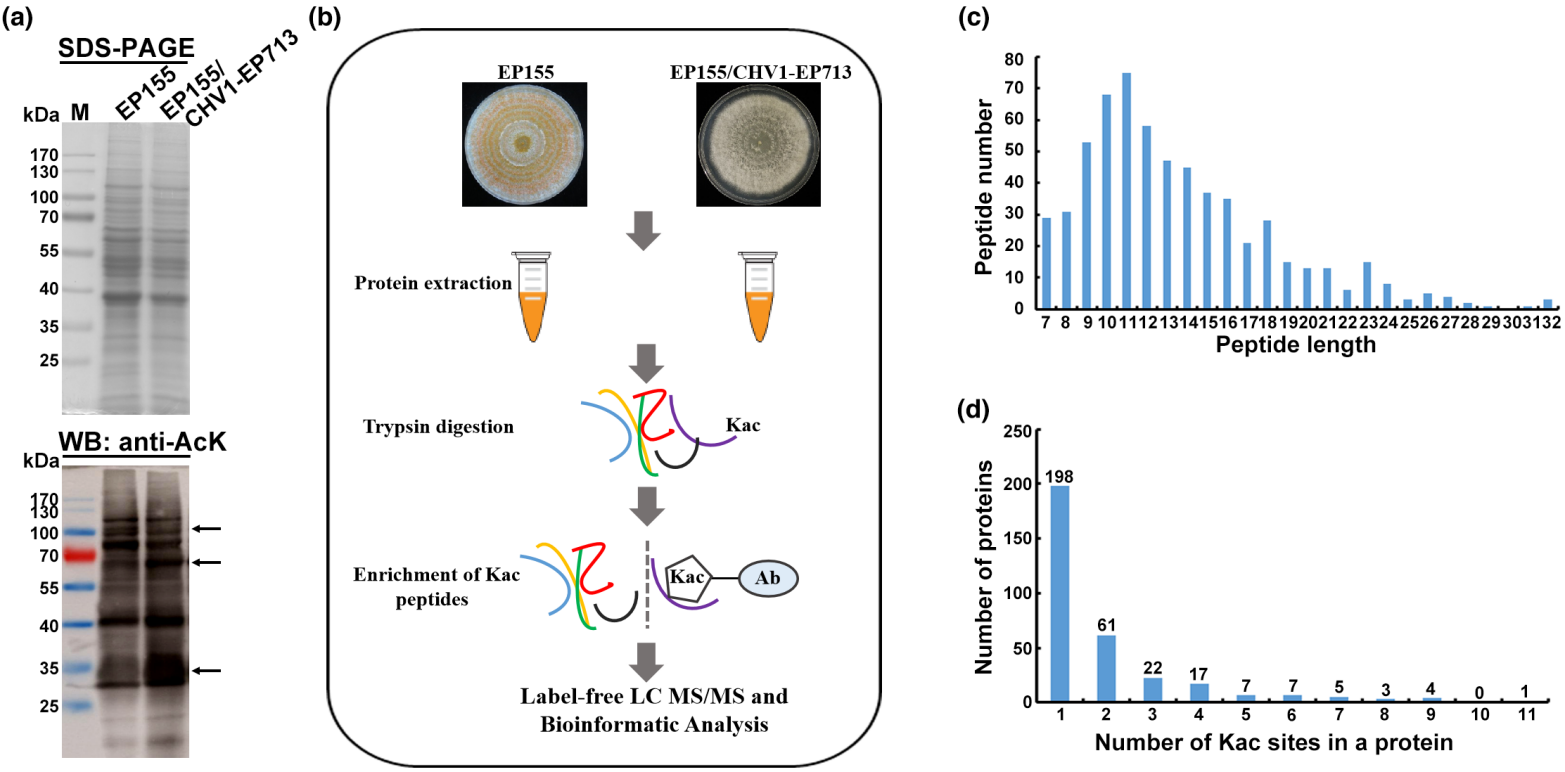
Proteome‐wide identification of lysine acetylation (Kac) sites and proteins in *Cryphonectria parasitica*. (a) Western blot (WB) analysis of acetylated proteins in *C*. *parasitica*. The SDS‐PAGE gel was stained or transferred to a polyvinylidene fluoride membrane and incubated with an anti‐acetyl‐lysine antibody. Total proteins were extracted from the wild‐type strain EP155 and its isogenic Cryphonectria hypovirus 1‐infected strain EP155/CHV1‐EP713. The arrow indicates differences in protein acetylation between two samples. (b) Schematic representation of the experimental procedures used in this study. (c) Distribution of acetylated peptide lengths. (d) Distribution of acetylated sites in acetylated proteins.

To investigate whether the global lysine acetylated protein level of *C*. *parasitica* was influenced by hypovirus infection, western blotting was performed using an anti‐acetyl‐lysine antibody. The acetylation patterns of total fungal proteins were different between the wild‐type strain EP155 and its isogenic Cryphonectria hypovirus 1 (CHV1)‐infected strain EP155/CHV1‐EP713, suggesting that the lysine acetylome of host proteins changed in response to hypovirus infection (Figure 1a). We therefore used a label‐free quantitative proteomic approach combined with immunoaffinity enrichment and liquid chromatography–tandem mass spectrometry (LC–MS/MS) to identify the acetylome of EP155 and EP155/CHV1‐EP713 (Figure 1b). To validate the MS data, the mass errors of all identified peptides were checked. The distribution of mass error was near 0 and most were less than 6 ppm, showing that the MS data conformed with the requirement (Figure S1). The results revealed 638 nonredundant Kac sites on 616 peptides, distributed in 325 unique proteins (Table S2). Most peptides were 7−18 amino acids in length (Figure 1c), which is consistent with the property of tryptic peptides. Among the 325 acetylated proteins, 198 contained one Kac site and most of the other 127 acetylated proteins contained two to four Kac sites (Figure 1d). A total of 75 and 65 proteins were distinctively acetylated in EP155 and EP155/CHV1‐EP713, respectively (Table S3). Further quantitative analysis of acetylated peptides was conducted between EP155 and EP155/CHV1‐EP713. A significant difference in acetylation is defined as changes in acetyl levels of more than 2‐fold and *p* values less than 0.05. The results showed that hypovirus infection induced 66 up‐regulated Kac sites in 43 proteins and 42 down‐regulated Kac sites in 37 proteins (Table S4). Based on western blot analysis and proteomics screening, hypovirus infection induced a wide change in global fungal proteins at the lysine acetylation level.

### 2.2 Conserved motif analysis of lysine acetylation sites

**FIGURE 2:**
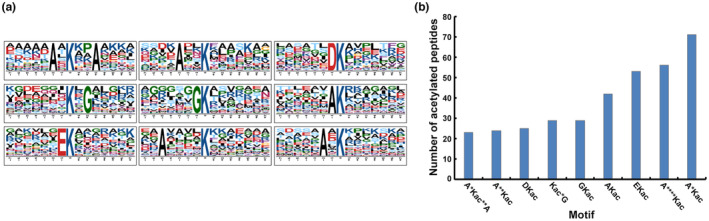
Properties of lysine acetylated peptides. (a) Motif analysis of the identified lysine acetylation sites. (b) Number of identified peptides in each conserved motif.

To identify possible motifs around the Kac sites, the sequences of all acetylated peptides were analysed to identify potential motifs using the Motif‐X software. The preferences for amino acid profiles were observed from positions −7 to +7 around the Kac sites. Nine enriched Kac site motifs were identified in the *C*. *parasitica* acetylome (Figure 2a and Table S5). In particular, motifs A*Kac, A****Kac, and EKac occupied the highest proportion (Kac represents acetylated lysine and * represents a random amino acid residue). The acetylated peptides with these motifs were 71, 56, and 53, which account for 20.2%, 16%, and 15% of all the identified peptides, respectively (Figure 2b and Table S4). Importantly, although most of the acetylation motifs identified in *C*. *parasitica* were also found in other eukaryotes (Chen et al., 2019; Guo et al., 2017; Liu et al., 2016; Zhu et al., 2016), the significantly conserved motifs (A*Kac, A****Kac, and Ekac) were rarely identified in other organisms. Because the acetylated peptides with the three motifs account for 51.2% of all identified peptides, it is likely that proteins with these motifs have some special function in *C*. *parasitica*.

### 2.3 Bioinformatics analysis of differentially lysine acetylated proteins

To investigate the functions and features of the differentially acetylated proteins, Gene Ontology (GO) functional classification and subcellular location prediction were performed. The first two distributed biological processes were metabolic process and cellular process on hypovirus infection (Figure S2). The results for the molecular function category showed that binding‐ and catalytic activity‐related proteins were preponderant because their percentages reached 49% and 43%, respectively. For the cellular component category, most of the differentially acetylated proteins belonged to the cell, organelle, and macromolecular complex. Subcellular location prediction results showed that differentially acetylated proteins were distributed in diverse subcellular locations, with the nucleus (31%), cytoplasmic matrix (29%), and mitochondria (27%) being prominent (Figure S3).

**FIGURE 3:**
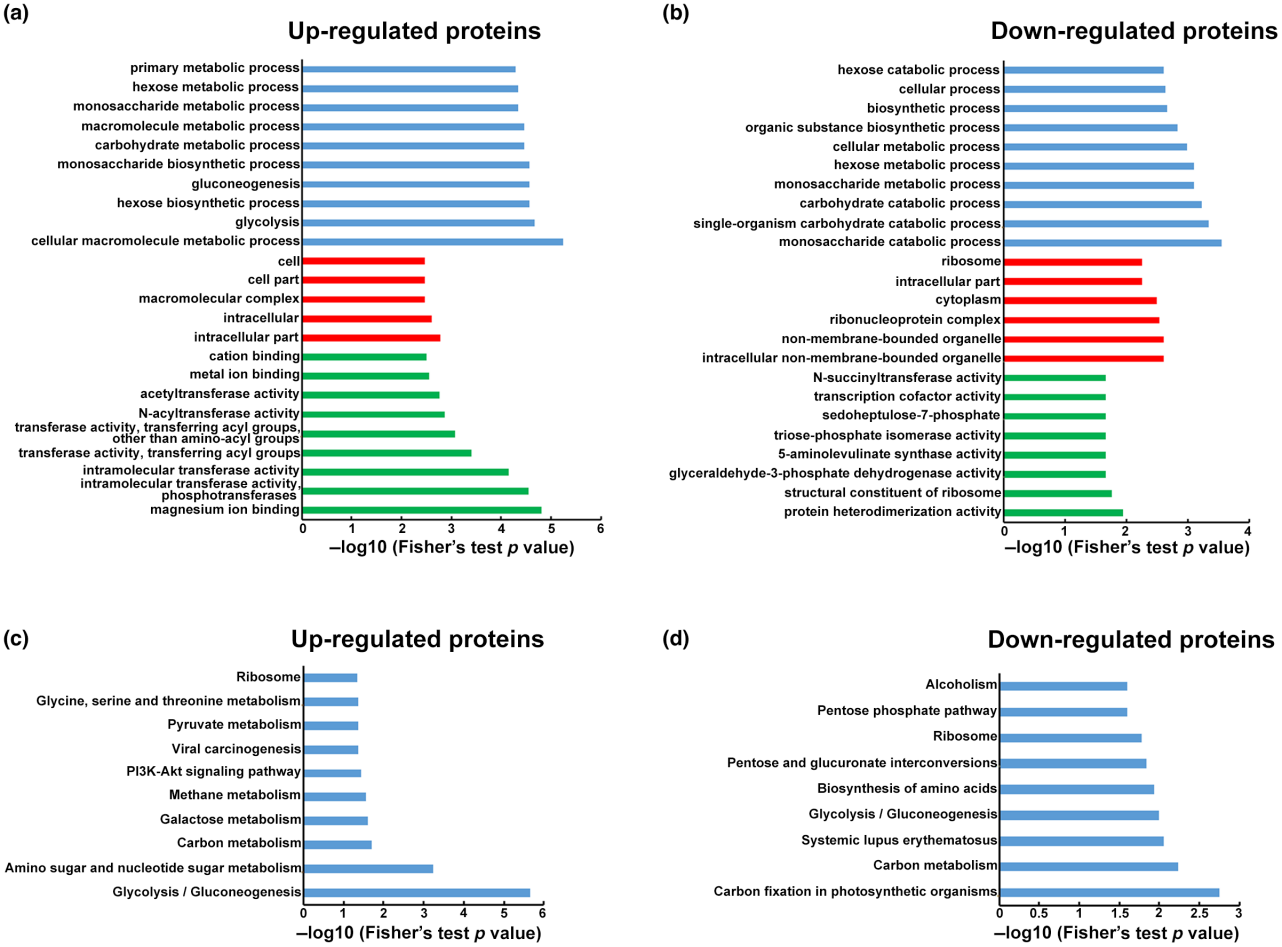
Enrichment analysis of the differentially expressed lysine acetylated proteins in *Cryphonectria parasitica* wild‐type strain EP155 and Cryphonectria hypovirus 1‐infected strain EP155/CHV1‐EP713. (a) GO‐based enrichment analysis of the up‐regulated proteins in terms of biological process (blue bars), cell component (red bars), and molecular function (green bars). (b) GO‐based enrichment analysis of the down‐regulated proteins in terms of biological process (blue bars), cell component (red bars), and molecular function (green bars). (c) KEGG pathway enrichment analysis of the up‐regulated proteins. (d) KEGG pathway enrichment analysis of the down‐regulated proteins. GO, Gene Ontology; KEGG, Kyoto Encyclopedia of Genes and Genomes.

To further elucidate the functionality of lysine acetylation of proteins regulated by hypovirus, enrichment analysis based on GO and Kyoto Encyclopedia of Genes and Genomes (KEGG) pathway was performed (Figure 3). In the up‐regulated proteins (Figure 3a and Table S6), metabolic processes and biosynthetic processes were enriched in the biological process category. Consistently, results for molecular function showed that many up‐regulated proteins were associated with binding and catalytic activity. In the cellular component category, intracellular space and macromolecular complex were significantly enriched. For down‐regulated proteins (Figure 3b and Table S7), monosaccharide and carbohydrate catabolism were enriched in the biological process category. Analysis based on molecular function showed that protein heterodimerization activity and structural constituent of ribosome were enriched. In cellular component category, intracellular nonmembrane bound organelle was enriched.

**FIGURE 4:**
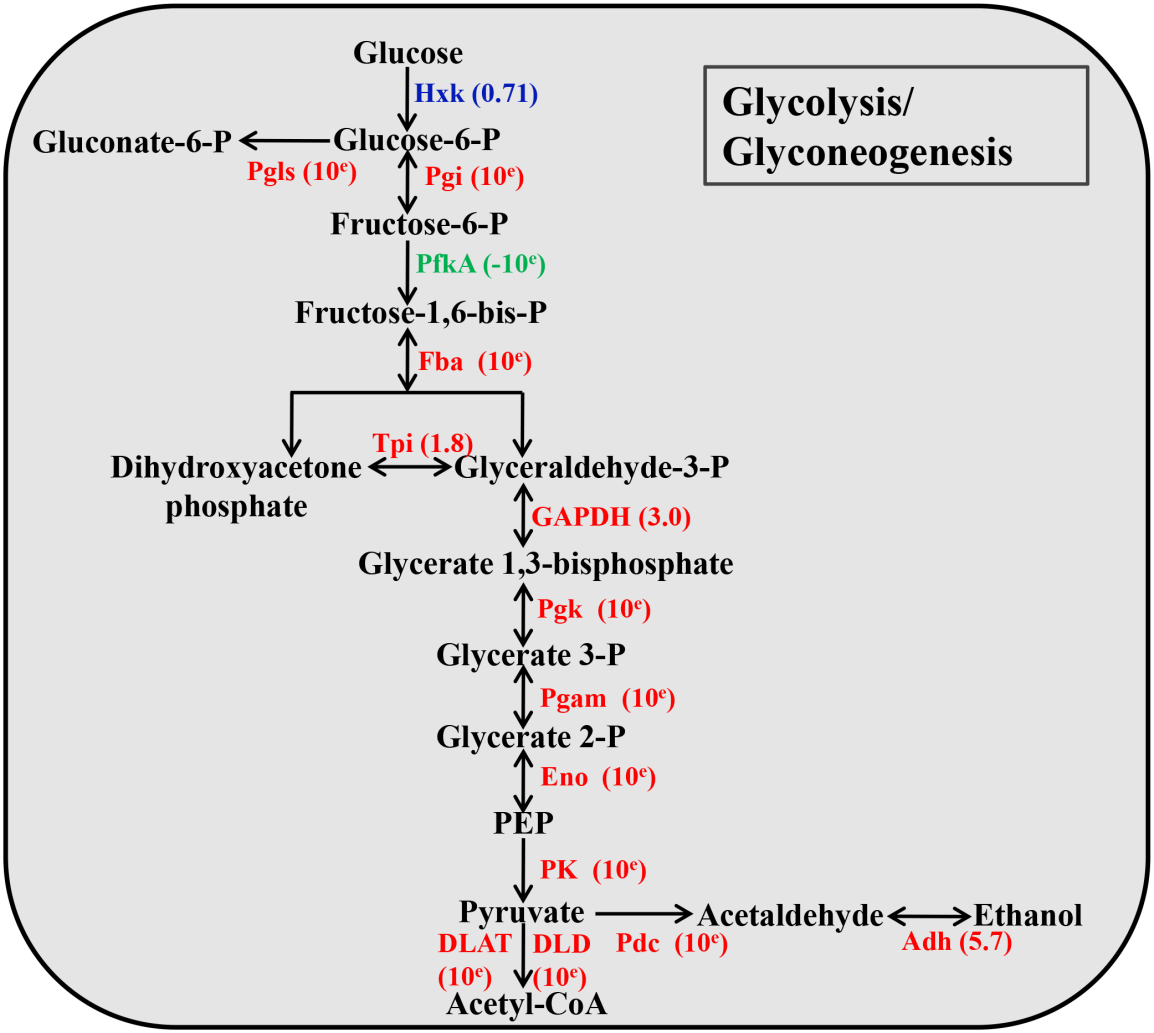
Schematic representation of the differentially expressed acetylated proteins involved in glycolysis or gluconeogenesis. *Cryphonectria parasitica* wild‐type strain EP155 was set as reference. The average change (Cryphonectria hypovirus 1‐infected strain EP155/CHV1‐EP713 versus EP155) were obtained from three independent experiments. A value of 10^e^ means that the protein acetylation appears only in EP155/CHV1‐EP713 and a value of −10^e^ means that the protein acetylation appears only in EP155. The proteins in red are up‐regulated and those in green are down‐regulated by hypovirus infection. Proteins in blue are expressed without significant differences. Hxk, hexokinase; Pgls, phosphogluconate dehydrogenase; Pgi, phosphoglucose isomerase; PfkA, phosphofructokinase; Fba, fructose‐bisphosphate aldolase; Tpi, triose‐phosphate isomerase; GAPDH, glyceraldehyde‐3‐phosphate dehydrogenase; Pgk, phosphoglycerate kinase; Pgam, phosphoglycerate mutase; Eno, phosphopyruvate hydratase; PK, pyruvate kinase; Pdc, pyruvate decarboxylase; Adh, alcohol dehydrogenase; DLAT, dihydrolipoamide acetyltransferase; DLD, dihydrolipoyl dehydrogenase.

The results of KEGG analysis revealed that the most enriched pathways for the up‐regulated proteins were glycolysis or gluconeogenesis, amino sugar and nucleotide sugar metabolism, and carbon metabolism (Figure 3c and Table S8). However, proteins with down‐regulated acetylation levels were enriched for carbon fixation in photosynthetic organisms (Figure 3d and Table S9). Changes in the most significantly enriched pathway, glycolysis or gluconeogenesis are shown in Figure 4. As indicated in the map, 13 proteins in this pathway were up‐regulated at the lysine acetylation level, including phosphogluconate dehydrogenase (Pgls), phosphoglucose isomerase (Pgi), fructose‐bisphosphate aldolase (Fba), triose‐phosphate isomerase (Tpi), glyceraldehyde‐3‐phosphate dehydrogenase (GAPDH), phosphoglycerate kinase (Pgk), phosphoglycerate mutase (Pgam), phosphopyruvate hydratase (Eno), pyruvate kinase (PK), pyruvate decarboxylase (Pdc), alcohol dehydrogenase (Adh), dihydrolipoamide acetyltransferase (DLAT), and dihydrolipoyl dehydrogenase (DLD). Based on these results, it appears that the differentially acetylated proteins are closely linked to metabolism in *C*. *parasitica*.

### 2.4 Hypovirus infection increases CpCS acetylation

**FIGURE 5:**
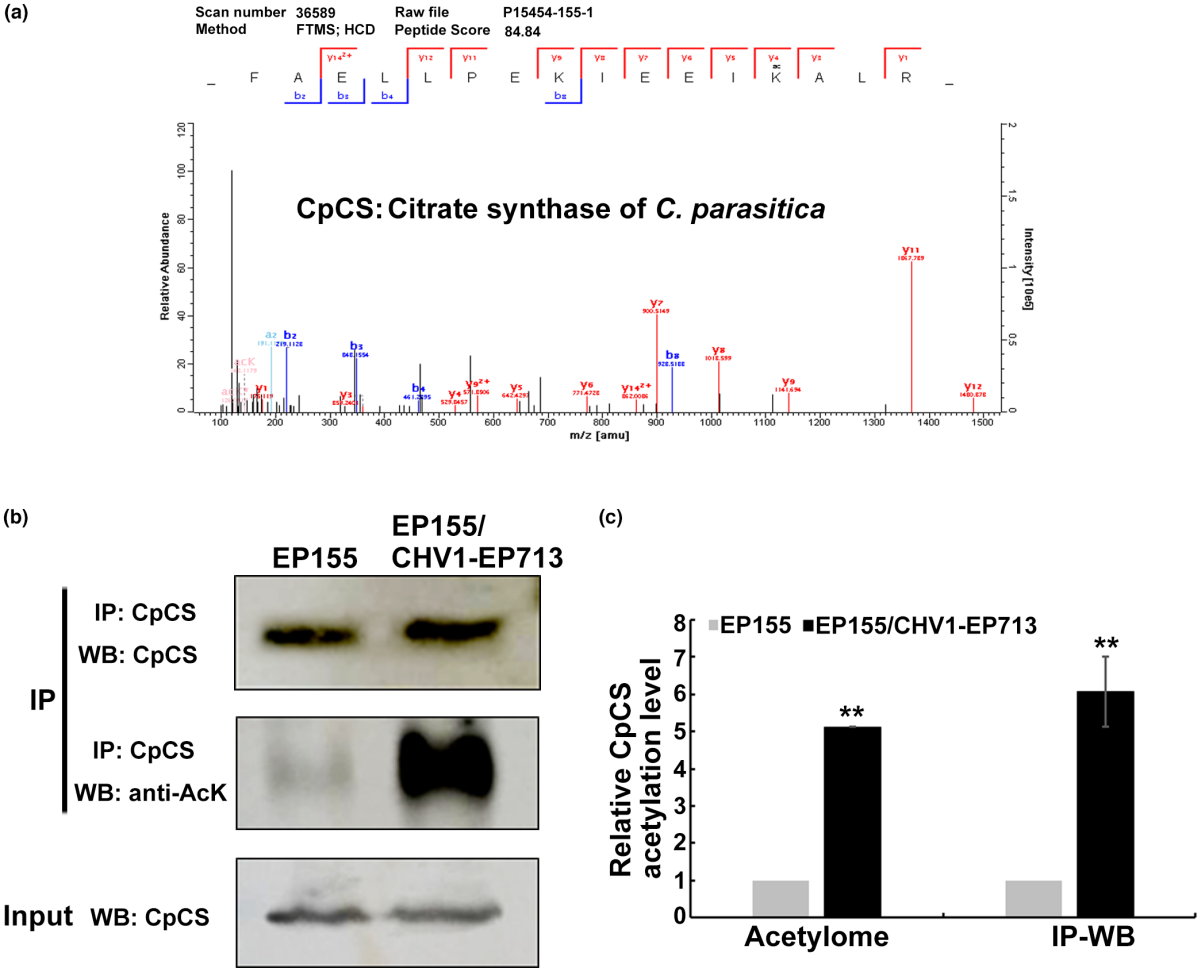
Verification of acetylated citrate synthase (CpCS) in *Cryphonectria parasitica* wild‐type strain EP155 and Cryphonectria hypovirus 1‐infected strain EP155/CHV1‐EP173. (a) MS/MS spectra of acetyl‐peptide. The peptide FAELLPEKIEEIK(ac)ALR is from CpCS. The acetylation site is indicated by ac. (b) CpCS was immunoprecipitated from *C*. *parasitica* cell lysate and detected by western blotting using an anti‐acetyl‐lysine antibody (anti‐Ack) or antibody specific for CpCS. Cell lysate without immunoprecipitation was used as the input. (c) Quantification of CpCS acetylation level. Average levels of CpCS acetylation were quantified using ImageJ and normalized relative to the value obtained in EP155, based on the acetylome data and western blotting (IP‐WB). The IP‐WB assays were repeated in triplicate. The error bars represent standard deviations. The asterisks (**) indicate a statistically significant difference from EP155 (*p* ≤ 0.01, *t* test).

MS data revealed that citrate synthase (CpCS) has an acetylated peptide (Figure 5a), and its acetylation level in EP155/CHV1‐EP713 was 5.1‐fold higher than that in EP155. To validate the acetylome results, immunoprecipitation and western blotting was performed using CpCS from EP155 and EP155/CHV1‐EP713 (Figure 5b,c). The results revealed no significant differences in the level of CpCS expression between the two samples, but the acetylation status of CpCS was up‐regulated 6.1‐fold in EP155/CHV1‐EP713. The result indicates that the acetylation level of CpCS was increased by hypovirus infection.

### 2.5 Lysine acetylation of CpCS is important for its function in *C*. *parasitica*

**FIGURE 6:**
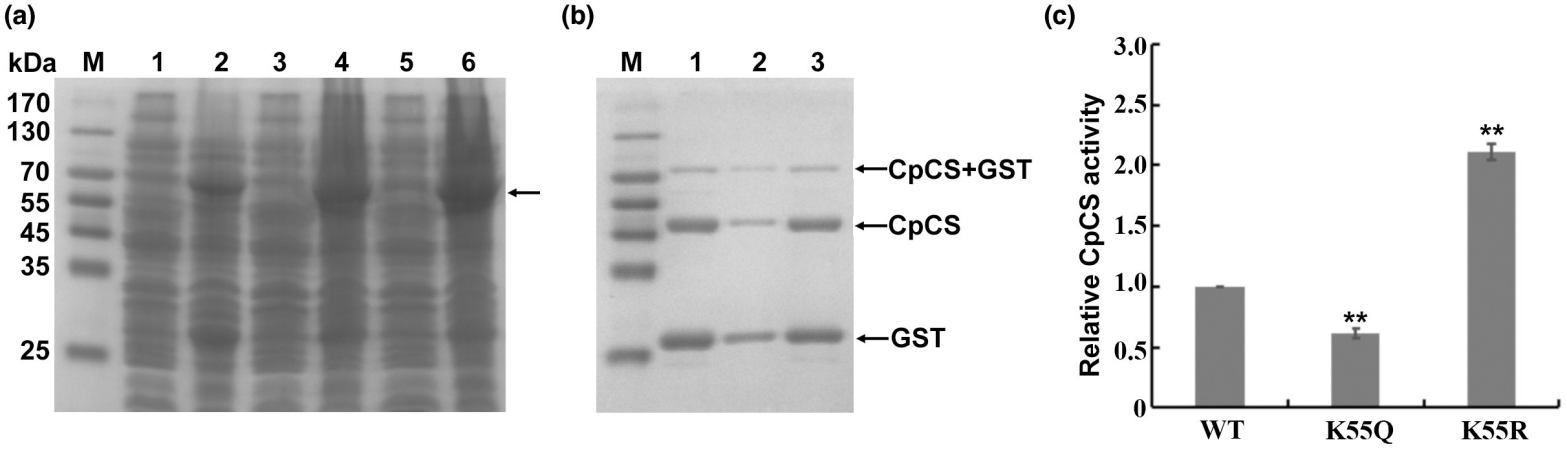
Expression, purification, and evaluation of enzyme activity of citrate synthase (CpCS) protein. (a) SDS‐PAGE analysis of CpCS protein expression. M, protein molecular mass marker; Lane 1, total protein from *Escherichia coli* BL21 (DE3)/pGEX‐4T‐1‐*CpCS* without IPTG induction; lane 2, total protein from *E*. *coli* BL21 (DE3)/pGEX‐4T‐1‐*CpCS* induced by IPTG; lane 3, total protein from *E*. *coli* BL21 (DE3)/pGEX‐4T‐1‐*CpCS* (K55Q) without IPTG induction; lane 4, total protein from *E*. *coli* BL21 (DE3)/pGEX‐4T‐1‐*CpCS* (K55Q) induced by IPTG; lane 5, total protein from *E*. *coli* BL21 (DE3)/pGEX‐4T‐1‐*CpCS* (K55R) without IPTG induction; lane 6, total protein from *E*. *coli* BL21 (DE3)/pGEX‐4T‐1‐*CpCS* (K55R) induced by IPTG. The arrow shows the induced CpCS protein. (b) SDS‐PAGE analysis of CpCS protein purification. Lane 1, purified CpCS; lane 2, purified CpCS (K55Q); lane 3, purified CpCS (K55R). The arrow shows the purified CpCS and glutathione‐*S*‐transferase (GST) protein. (c) Citrate synthase activity of purified CpCS (wild‐type, WT), mutant CpCS (K55Q), and mutant CpCS (K55R). Enzyme activity was measured by using 5,5′‐dithiobis‐(2‐nitrobenzoic acid) (DTNB) and the same amount of protein. The activity of WT was set as 1. The error bars represent standard deviations from three independent experiments. The asterisks (**) indicate a statistically significant difference from WT (*p* ≤ 0.01, *t* test).

According to the result shown in Figure 5a, a reliable acetylation site (lysine‐55) was identified on CpCS. Additional amino acid sequence alignment results showed that lysine‐55 of CpCS was highly conserved in *C*. *parasitica* orthologues, indicating that this residue may be necessary for the conserved function of CpCS (Figure S4). Recombinant CpCS was overexpressed in *Escherichia coli* (Figure 6a) and purified to test whether the lysine‐55 acetylation would affect the enzymatic activity of CpCS. The modified residue was mutated to glutamine (K55Q) and arginine (K55R) to mimic the acetylation and deacetylation state on lysine as previous studies, respectively (Guan et al., 2021; You et al., 2019). The successful construction of both mutant plasmids was verified by DNA sequencing results (Figure S5). Compared with wild‐type (WT) protein, CpCS‐K55Q displayed significantly reduced enzyme activity. Meanwhile, the replacement of lysine‐55 with arginine resulted in a decrease in the acetylation level of CpCS and a significant increase in its enzyme activity (Figures 6 and S6). These data indicate that the acetylation of lysine‐55 is critical for CpCS enzyme activity in vitro.

**FIGURE 7:**
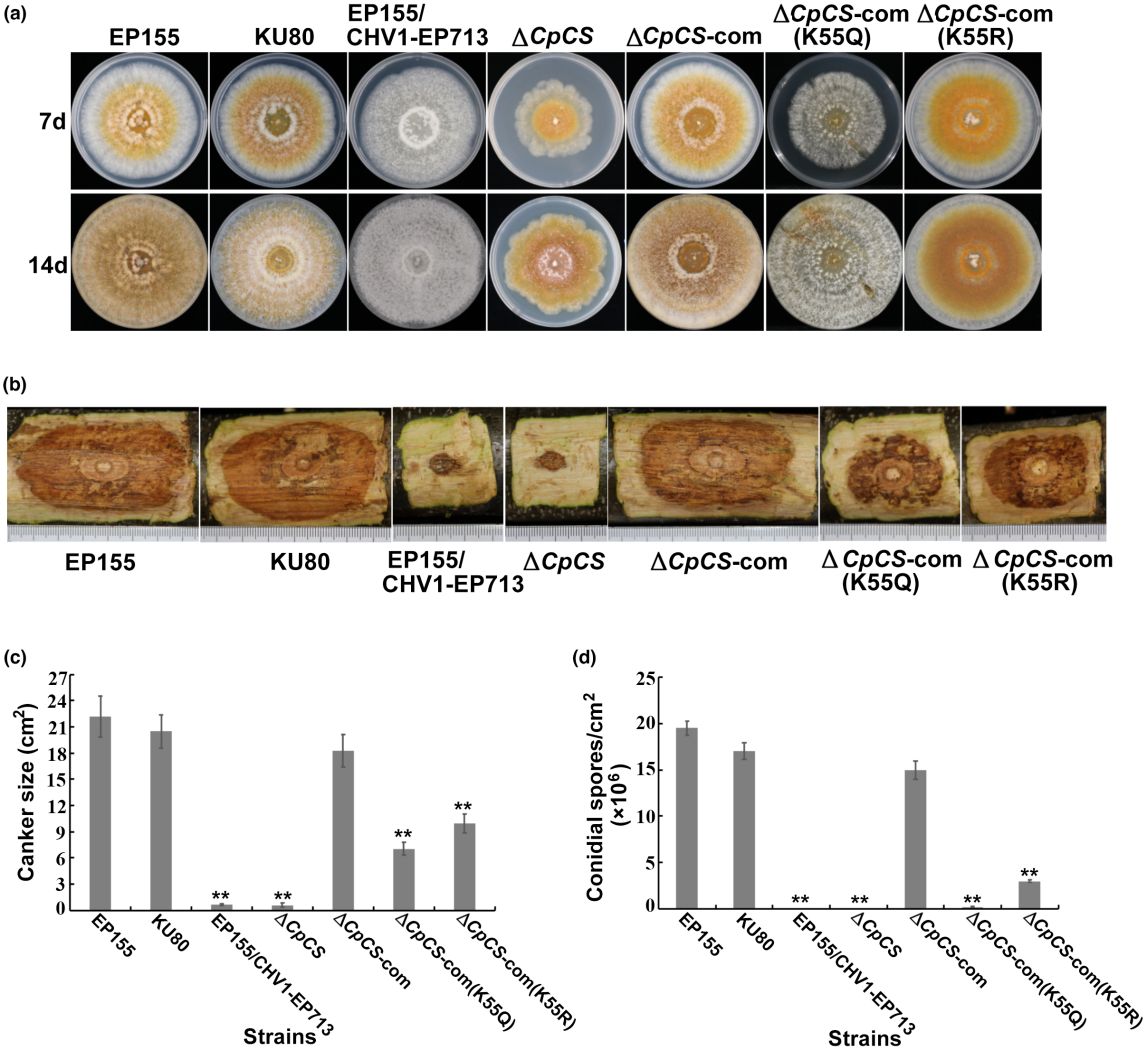
Analysis of phenotypes, virulence, and sporulation of *CpCS* deletion mutants. (a) Mutant colony morphologies on potato dextrose agar plates. Photographs were taken 7 and 14 days after inoculation. The strains indicated are wild‐type EP155, its Cryphonectria hypovirus 1‐infected isogenic strain EP155/CHV1‐EP713, the gene disruption strain KU80, *CpCS* deletion strain ∆*CpCS*, the complementation strain ∆*CpCS*‐com, and the mutant complementation strains ∆*CpCS*‐com (K55Q) and ∆*CpCS*‐com (K55R). (b) Cankers induced by the tested strains on dormant stems of Chinese chestnut. The inoculated stems were kept at 26°C and cankers were measured and photographed 25 days after inoculation. (c) Canker size measurements of the tested strains. (d) Sporulation levels of the indicated strains. Spores were counted on day 14. The error bars represent standard deviations from three independent experiments. The asterisks (**) indicate a statistically significant difference from EP155 (*p* ≤ 0.01, *t* test).

To further examine the potential function of lysine acetylation on CpCS in *C*. *parasitica*, we first constructed the *CpCS* null mutant using replacement with the hygromycin‐resistance gene (*hph*). The single‐spored transformants were screened by PCR and confirmed by Southern blotting (Figure S7). When the *CpCS* null mutant was grown on a potato dextrose agar (PDA) plate, it displayed a defect in growth rate and had an irregular colony margin relative to the wild‐type strain EP155 and parent strain KU80 (Figure 7a). In addition, the deletion of *CpCS* resulted in a remarkable reduction in virulence and sporulation. The complemented strain ∆*CpCS*‐com restored the abnormal phenotypes compared with the parent strain (Figure 7b–d). Next, the sequencing‐confirmed *CpCS* lysine‐55 mutated genes were introduced into the *CpCS* null mutant using pCPXG418‐*CpCS* (K55Q) and pCPXG418‐*CpCS* (K55R). Compared with the wild‐type and ∆*CpCS*‐com strains, the site‐specific mutant ∆*CpCS*‐com (K55Q) grew much more slowly on PDA plates and exhibited reduced pigment, virulence, and conidial spores. In contrast, the colony morphology and growth rate of the site‐specific mutant ∆*CpCS*‐com (K55R) were similar to the wild‐type and ∆*CpCS*‐com strains. However, obvious decreases in sporulation and virulence occurred in the mutant. Mutation of K55R had less impact on virulence and sporulation than mutation of K55Q (Figure 7). This finding suggests that the acetylation of lysine‐55 is important in regulating CpCS function in vivo.

## 3 DISCUSSION

Protein lysine acetylation is one of the most ubiquitous posttranslational modifications that fine‐tunes the major cellular processes of many life forms (Narita et al., 2019), but little is known about how this modification functions in fungi. In this study, the lysine acetylome of *C*. *parasitica* was determined to examine the effects of CHV1 infection on protein lysine acetylation profiles. To our knowledge, this study is the first comprehensive analysis of lysine acetylated proteins in response to a hypovirus infection covering the entire acetylome of a fungus.

A total of 616 unique acetylation peptides, encompassing 638 high‐confidence Kac sites, were identified from 325 *C*. *parasitica* proteins. Most proteins were acetylated at a single lysine site. However, some acetylated proteins, including histone H2B (11 sites), GAPDH (nine sites), Eno (nine sites), acetyl‐CoA acetyltransferase (ACAT1, nine sites), and PK (nine sites), contained multiple modification sites. These results suggest that lysine acetylation is abundant in these proteins. Emerging evidence has shown that nearly all major metabolic enzymes undergo acetylation, demonstrating the existence of a common mechanism of acetylation involved in metabolic regulation (Chen et al., 2020; Huang et al., 2014; Wang et al., 2010). Consistent with these findings, a large proportion of metabolic enzymes involved in central metabolism were found to be acetylated, such as enzymes involved in glycolysis or gluconeogenesis, the TCA cycle, and fatty acid metabolism, implying that lysine acetylation may regulate cellular metabolic processes in *C*. *parasitica*. Additionally, protein acetylation occurs in proteins involved in the ribosome, mitochondrion, and protein translation and folding, which is consistent with previous studies (Li, Lv, et al., 2016). In this study, some modification enzymes, including methyltransferase (protein ID: 226763, 263,282) and *N*‐acetyltransferase (protein ID: 103287, 247,896, 248,339, 258,510), were found to be acetylated. In agreement with these observations, AflO—a key *O*‐methyltransferase for aflatoxin synthesis in *A. flavus*—was found to be acetylated at K241 and K384. Lysine acetylation plays a vital role in the regulation of the enzymatic activity of AflO (Yang et al., 2019). Some proteomic studies have also observed lysine acetyltransferases among acetylated proteins, which can acetylate specific lysine residues in histones or other proteins (Li, Lv, et al., 2016; Vogelauer et al., 2012). Notably, acetyltransferases were shown to play a critical role in morphogenetic hyphae growth, biofilm formation, drug resistance, and virulence of fungi (Dubey et al., 2019; Zhang et al., 2021). Overall, these findings suggest that lysine acetylation is widely distributed in *C*. *parasitica* and may play an important regulatory role in diverse biological processes.

Previous studies have reported that human Borna disease virus infection alters the acetylome of host cells to increase energy levels and transporters (Liu et al., 2015). Comparative proteomic analysis of lysine acetylation in fish CIK cells suggested that cellular metabolism was greatly altered due to grass carp reovirus (Guo et al., 2017). In silkworm cells, baculovirus infection globally impacted the acetylome of host cells. A total of 6.96% and 14.8% of 431 quantified proteins were significantly up‐ or down‐regulated, respectively, and were mostly involved in metabolism and biosynthesis (Hu et al., 2018). Recently, Murray et al. (2018) reported that protein lysine acetylation regulates both virus replication and antiviral defence by switching on and off protein functions. To evaluate the impact of fungal viruses on host protein acetylation, intensive proteomic quantification analysis was used to generate lysine acetylation profiles of *C*. *parasitica* with or without CHV1 infection. Consistent with other reports, the lysine acetylome of host proteins was found to be changed in response to hypovirus infection (Figure 1a). Furthermore, many acetylated proteins affected by hypovirus infection were found to be involved in metabolic processes according to GO classification (Figure S2). KEGG pathway enrichment analysis revealed significant enrichment of up‐regulated proteins in glycolysis or gluconeogenesis, amino sugar and nucleotide sugar metabolism, and carbon metabolism (Figure 3c). Notably, most glycolytic enzymes from the CHV1‐infected strain had higher levels of acetylation compared with enzymes from the wild‐type strain, suggesting that this modification may modulate enzyme activity (Figure 4). GAPDH was one of the differentially acetylated enzymes. GAPDH is an obligatory enzyme in glycolysis. GAPDH acetylation increases its activity and promotes cell proliferation and tumour growth (Li et al., 2014). Pgk is a crucial enzyme in glycolysis. The acetylation of Pgk1 (K323) reportedly promotes its enzyme activity and cancer cell metabolism (Hu et al., 2017). PK is involved in the last step of glycolysis. A recent study found that increasing PKM2 hyperacetylation may be closely related to hepatotoxicity by increasing its activity and intracellular lactate concentration (Na et al., 2021). In light of the critical role of glycolysis in cellular energy production, it is reasonable to speculate that CHV1 perturbs the energy balance by regulating the acetylation of metabolic enzymes. Consistent with our findings, a metabolomic analysis in another study found that CHV1 infection increased the metabolic rate of *C*. *parasitica*, resulting in greater glucose depletion (Dawe et al., 2009). Nevertheless, more research is needed on how hypovirus infection alters global cellular acetylation dynamically.

Comparison of this acetylome with previous reported proteomic results (Wang et al., 2013, 2014) would be helpful for evaluating the relationship between protein level and protein acetylation intensity. When we compared the differentially acetylated proteins and differentially expressed general proteins based on the same samples, only five proteins were found differentially expressed both in the protein level and acetylation level. Furthermore, we found that the acetylation levels of the three proteins enolase (protein ID: 103155), glyceraldehyde‐3‐phosphate dehydrogenase (protein ID: 101684), and alcohol dehydrogenase‐1 (protein ID: 106275) increased while the total protein levels decreased. The acetylation level and total protein level of eukaryotic translation initiation factor 5A (protein ID: 340620) and peptidyl‐prolyl *cis*‐*trans* isomerase (protein ID: 277770) were both decreased. According to this comparison, most proteins showing abundant changes were not lysine acetylated. Additionally, most of the differentially acetylated proteins showed no significant changes in protein level. This discrepancy between protein expression abundance and protein acetylation levels indicates that the protein acetylation change by the CHV1 infection is mostly independent of protein quantity.

Viral proteins are also reported to be acetylated by the host acetyltransferase, which may be relevant for viral replication (Kumar et al., 2020). For example, human immunodeficiency virus Tat acetylation serves as a critical step in its transcriptional activity (Ott et al., 2004). Lysine acetylation regulates the activity of influenza virus proteins, such as NP (K77, K113, and K229) and NS1 (K108), which are important for viral replication and growth (Giese et al., 2017; Ma et al., 2020). Murray et al. (2018) demonstrated that the acetylation of human cytomegalovirus transcriptional activator pUL26 inhibits virus production. Recently, 39 Kac sites were identified in 22 acetylated Bombyx mori nucleopolyhedroviral proteins (Hu et al., 2018). In this study, no viral proteins were found to be acetylated based on the acetylome of EP155/CHV1‐EP713. It is possible that the regulatory mechanism of mycoviruses differs significantly from that of animal viruses. However, how posttranslational modification functions in mycovirus replication remains unclear.

Among all metabolic pathway enzymes, TCA cycle enzymes are acetylated and involved in the final common oxidative pathway for carbohydrates, fats, and amino acids in other organisms (Wang et al., 2010). This study found that six proteins involved in the TCA cycle were acetylated, suggesting a potentially conserved role of acetylation in regulating the pathway. Citrate synthase catalyses the first reaction of the TCA cycle, playing an important role in central metabolism and amino acid biosynthesis (Weitzman, 1980). The data of this study show that CpCS contains an acetylated peptide, and its acetylation was 5.1‐fold greater in EP155/CHV1‐EP713 than in EP155. We hypothesized that lysine acetylation may be important for regulating fungal phenotypic traits by affecting CpCS function. To investigate the role of lysine acetylation in CpCS enzyme activity, the protein was expressed in *E*. *coli* and proteins containing point mutations were constructed. While the K55Q mutation decreased the enzyme activity of CpCS, the K55R mutation increased it (Figure 6). Consistently, the enzyme activity of eukaryotic type I citrate synthase was reported to be significantly reduced when acetylation increased (Cui et al., 2017). A similar observation was reported in *E*. *coli* type II citrate synthase (Venkat et al., 2019). Furthermore, in this study, *CpCS* was deleted and point mutant strains were constructed to investigate the role of lysine acetylation on the growth, sporulation, and virulence of *C*. *parasitica*. K55Q and K55R mutants showed a significant decrease in sporulation and virulence (Figure 7). Thus, it is proved that the lysine acetylation of CpCS could affect the enzyme activity and fungal phenotypic traits in *C*. *parasitica*.

In summary, these results represent the first extensive data on lysine acetylation in *C*. *parasitica* with or without CHV1 infection. Although these findings improve our understanding of fungal protein regulation by a hypovirus from a lysine acetylation perspective, further experiments will be needed to investigate this interesting phenomenon.

## 4 EXPERIMENTAL PROCEDURES

### 4.1 Fungal strains and growth conditions

The *C*. *parasitica* WT strain EP155 (ATCC 38755), its isogenic strain EP155/CHV1‐EP713 (EP155 infected with synthetic hypovirus CHV1‐EP713, ATCC 52571) (Chen et al., 1994), and a highly efficient gene disruption strain KU80 (∆*ku80* of EP155) (Lan et al., 2008) were used in this study. For morphological characterization and DNA and RNA extraction, the strains were grown on PDA at 26°C with a 12 h light/dark cycle (Hillman et al., 1990). For protein extraction, the strains were cultured in EP complete liquid medium for 3 days with shaking at 100 rpm (Puhalla & Anagnostakis, 1971).

### 4.2 Protein extraction

Fungal proteins were extracted based on a protocol modified from Wang et al. (2014). Briefly, fungal mycelia were ground into powder in liquid nitrogen, resuspended in five‐fold volumes of prechilled trichloroacetic acid:acetone (1:9), incubated at −20°C overnight, and centrifuged at 7000 *g* for 15 min at 4°C. The supernatant was discarded and the precipitate was washed three times with precooled acetone. The precipitate was air dried and resuspended in lysis buffer (8 M urea, 50 mM Tris–HCl pH 8.0, 80 mM dithiothreitol, 2% protease inhibitor). The suspension was ultrasonicated 10 times at 100 W (for 10 s, with 10 s intervals) and centrifuged at 15,000 *g* for 20 min. Protein concentration was quantified with the Bradford assay (Bio‐Rad) and the protein was stored at −80°C until use.

### 4.3 Trypsin digestion and affinity enrichment of acetylated proteins

Protein digestion was performed with trypsin, as described, with some minor modifications (Guo et al., 2017). In brief, the protein sample was diluted with 100 mM NH_4_CO_3_ to a urea concentration of about 1 M. Then, trypsin (Promega) was added at 1:50 trypsin: protein mass ratio overnight at 37°C. Finally, the peptides of each sample were desalted on the C18‐SD extraction disk cartridge and vacuum‐dried.

Samples of three biological triplicates were subjected to lysine‐acetylated peptide enrichment (Rappsilber et al., 2007). The tryptic peptides were dissolved in NETN buffer (100 mM NaCl, 1 mM EDTA, 50 mM Tris–HCl pH 8.0, 0.5% Nonidet 40) and incubated with anti‐acetyl‐lysine antibody conjugated to agarose (Cell Signalling Technology) overnight at 4°C. The beads were washed four times with NETN buffer and two times with deionized water. The acetylated peptides were eluted from the beads by washing two times with 0.15% trifluoroacetic acid. The resulting peptides were cleaned with C18 STAGE tips according to the manufacturer's instructions, and then subjected to LC–MS/MS analysis.

### 4.4 LC–MS/MS analysis

The enriched peptides were analysed with the EASY‐nLC1000 system (Thermo Finnigan) connected to a Q Exactive mass spectrometer (Thermo Finnigan). Briefly, peptide samples were dissolved in 0.1% formic acid, directly loaded onto a reverse‐phase precolumn (Thermo EASY column SC200, 150 μm × 100 mm). A reverse‐phase analytical column (Thermo EASY column SC001 traps, 150 μm × 20 mm) was used for peptide separation, as described previously (Li, Sun, et al., 2016). The resulting peptides were evaluated with the Q Exactive mass spectrometer for 120 min. The mass spectrometer was operated in positive ion mode. MS data were acquired using a data‐dependent procedure, dynamically choosing the most abundant precursor ions from the survey scan (350–1800 *m/z*) for high‐energy collision dissociation fragmentation. Survey scans were acquired at a resolution of 70,000 at *m/z* 200 and the resolution for high‐energy collision dissociation spectra was set to 17,500 at *m/z* 200. Automatic gain control was used to prevent overfilling of the ion trap and 5 × 10^4^ ions were accumulated for generating the MS/MS spectra. Each LC–MS/MS analysis was repeated three times to reduce technical variation.

### 4.5 Data analysis

The resulting MS/MS data were processed using the MaxQuant search engine (v. 1.5.2.8). Tandem mass spectra were searched against the *C*. *parasitica* database (http://genome.jgi‐psf.org/Crypa2/Crypa2.home.html) concatenated with the reverse decoy database. Trypsin was specified as a cleavage enzyme, allowing up to four missing cleavages. Mass tolerance for precursor ions was set at 20 ppm in the first search and 6 ppm in the main search. Mass tolerance for fragment ions was set as 0.02 Da. Carbamidomethyl on Cys was specified as a fixed modification, and oxidation on Met, acetylation on Lys, and acetylation on protein N‐terminal were specified as variable modifications. False discovery rate thresholds were specified at 1%. All the other parameters in MaxQuant were set to default values. The quantitative value of each acetylated peptide was calculated based on intensity information derived from LC–MS data as previously described (Guo et al., 2017). Then the ratio of the average value between the two samples was calculated.

### 4.6 Bioinformatics analysis

Proteins were classified using GO database annotation (Hulsegge et al., 2009), based on three categories: biological process, cellular component, and molecular function. KEGG (Moriya et al., 2007) was used to annotate protein pathways. The WoLF PSORT software was used to analyse subcellular localization (Horton et al., 2007). Functional enrichment analysis was performed using the DAVID Bioinformatics Resources 6.7 (Huang et al., 2007). The motif‐*x* software was used to analyse the model of sequences constituted with amino acids in specific positions of acetyl‐15‐mers (seven amino acids upstream and downstream of the site) in all protein sequences (Chou & Schwartz, 2011). Functional interaction network analysis was performed using the publicly available program STRING (http://string‐db.org/).

### 4.7 Immunoprecipitation and western blotting

To verify the acetylation of CpCS, immunoprecipitation was performed as reported (Mo et al., 2015). Equal amounts (50 μg total protein) were incubated with 1 μg of the anti‐CpCS antibody (preserved in our laboratory) in a microcentrifuge tube overnight at 4°C. Then, the antibody–lysate sample was added to 20 μL of protein A/G plus agarose in the spin column with gentle end‐over‐end mixing for 1 h. The conjugated resin was washed three times with the immunoprecipitation lysis/wash buffer to remove unbound proteins. Bound proteins were boiled with SDS loading buffer for 10 min, separated using 12% SDS‐PAGE, and transferred to a polyvinylidene fluoride membrane (Millipore) using the Hoefer TE 77 semidry transfer unit (Hoefer). For western blotting, the membranes were blocked with 5% nonfat milk in Tris‐buffered saline with 0.1% Tween 20 (TBST) for 1 h. Primary anti‐acetyl‐lysine antibody (Cell Signalling Technology) or CpCS antibody were diluted at 1:1000 in TBST. The membranes were incubated overnight at 4°C with the antibodies, washed with TBST, incubated with a horseradish peroxidase‐conjugated secondary antibody (1:5000) for 1 h at 37°C, and detected according to the manufacturer's instructions.

### 4.8 Measurement of CpCS activity

For expressing recombinant *Cp*CS in *E. coli*, a cDNA fragment of *CpCS* was amplified by PCR using primers CpCS‐Ex‐F and CpCS‐Ex‐R (Table S1). The PCR product was cloned into the prokaryotic expression vector pGEX‐4T‐1 (GE Healthcare) to generate pGEX‐4T‐1‐*CpCS*. Transformed *E*. *coli* BL21 (λDE3)/pGEX‐4T‐1‐*CpCS* were induced with isopropyl‐β‐d‐thiogalactopyranoside (IPTG), lysed, and purified using a GST‐Sefinose resin column (Sangon Biotech). The quality of recombinant proteins was analysed by SDS‐PAGE. CpCS was further concentrated by ultrafiltration using a 30,000 molecular weight cut‐off (MWCO) concentrator (Millipore) in storage buffer (50 mM Tris–HCl pH 8.0). Protein concentrations were measured using the Bradford protein assay kit (Beyotime Institute of Biotechnology). pGEX‐4T‐1‐*CpCS* was used to generate the K55Q and K55R mutant plasmid using the Mut Express II Fast Mutagenesis Kit V2 (Vazyme Biotech). Then mutated CpCS (K55Q) and CpCS (K55R) proteins were prepared with the mutated plasmid pGEX‐4T‐1‐*CpCS* (K55Q) and pGEX‐4T‐1‐*CpCS* (K55R), respectively. Quantitative determination of CpCS enzymatic activity was performed as described previously (Lemos et al., 2003), using purified CpCS (WT), mutant CpCS (K55Q) or CpCS (K55R) proteins. Enzyme activities were obtained by the difference in absorbance between the samples and blanks and were given as units (U) based on the extinction coefficient of the product.

### 4.9 Generation of fungal mutants

*CpCS* deletion mutants were generated using the KU80 strain and homologous recombination method (Lan et al., 2008). Sequences upstream and downstream of *CpCS* in the genomic DNA were amplified using the primers listed in Table S1. The hygromycin B resistance (*hph*) marker was amplified using plasmid pCPXHY2 as template with primers Hyg‐F and Hyg‐R (Chen et al., 2011). The upstream and downstream DNA sequences and *hph* were joined by overlapping PCR. The purified PCR products were transformed into KU80 protoplasts. Transformants were selected on PDA supplemented with hygromycin. Putative *CpCS* deletion mutants were confirmed using PCR (primers CpCS‐all‐F and CpCS‐all‐R; Table S1) and Southern blotting, and purified to nuclear homogeneity by single‐spore isolation (Sambrook & Russell, 2001). To generate the complementation strain Δ*CpCS*‐com, the complete open reading frame of *CpCS* (including the promoter sequence) was amplified and inserted into the transformation vector pCPXG418 (containing the G418 resistance marker) (Chen et al., 2011), generating the wild‐type gene complementation plasmid pCPXG418‐*CpCS*. The resulting plasmid was used for the generation of the K55Q and K55R mutant plasmids using the Mut Express II fast mutagenesis kit V2 (Vazyme Biotech). Each mutant plasmid was confirmed by DNA sequencing. pCPXG418‐*CpCS*, pCPXG418‐*CpCS* (K55Q) and pCPXG418‐*CpCS* (K55R) were transformed into the knockout strain Δ*CpCS*, respectively. Complementation was selected on PDA supplemented with hygromycin and G418, and confirmed by PCR with primers CpCS‐all‐F and CpCS‐all‐R. All primer sequences used in the construction and verification of fungal mutants were shown in Table S1.

### 4.10 Virulence assay

For the fungal virulence analysis, dormant stems of Chinese chestnut (*Castanea mollissima*) were inoculated and incubated in a plastic bag at 26°C to allow lesion development, as described (Liao et al., 2012). Canker size was observed and analysed 25 days after inoculation. The infection experiments were repeated three times for each fungal strain.

## Supporting information


**Figure S1** The distribution of peptides mass error (ppm) based on *m*/*z* of 774 identified acetylation peptides

**Figure S2** Gene Ontology (GO) classification analysis of the differentially expressed acetylated proteins

**Figure S3** Subcellular location prediction of the differentially expressed acetylated proteins

**Figure S4** Amino acid sequence alignment of CpCS and its orthologues: *Cryphonectria parasitica* CpCS (JGI 104411), *Aspergillus flavus* AfCS (XP_041146416.1), *Neurospora crassa* NcCS (XP_956898.1), *Saccharomyces cerevisiae* ScCit1 (NP_014398.1), *Arabidopsis thaliana* AtCS (NP_001327513.1), and *Homo sapiens* HsCS (NP_004068.2). The alignment was made using MegAlign in ClustalW. The red arrow represents lysine 55 of CpCS

**Figure S5** DNA sequencing results of *CpCS* mutations on the pGEX‐4T‐1‐*CpCS* vector

**Figure S6** Lysine 55 mutation reduces the acetylation of CpCS. Experiments were replicated three times and representative results are shown. (a) SDS‐PAGE of samples. (b) The acetylation levels of the two proteins were determined by western blotting using an anti‐acetyl‐lysine antibody. (c) The acetylation levels of CpCS were quantified from the western blot using ImageJ and normalized relative to the value obtained with the wild type (WT) (lane 1). The asterisk indicates a statistically significant difference from lane 1 (*p* ≤ 0.05, *t* test)

**Figure S7** Southern blot analysis of *CpCS* knockout mutants. (a) Schematic representation of *CpCS* gene replacement approach. Fragment on the left arm (probe 1) and fragment on the *hph* gene (probe 2) were used in the Southern blot analysis to distinguish the fragment size of the wild‐type strain and *CpCS* knockout mutants. Scale bar = 1 kb. (b) Southern blot analysis of *CpCS* knockout mutants. Fungal total DNAs were digested with XhoI and separated in a 1.0% agarose gel by electrophoresis, then blotted using probe 1 and probe 2, respectively. Fragment sizes are indicated in the figure margins

**Table S1** Primers used in this study

**Table S2** Origin data of acetylation lysine sites

**Table S3** Distinct Kac sites in EP155 and EP155/CHV1‐EP713

**Table S4** Differential Kac proteins

**Table S5** The most significant Kac motifs in sequence

**Table S6** GO annotation of up‐regulated Kac proteins

**Table S7** GO annotation of down‐regulated Kac proteins

**Table S8** KEGG pathway of up‐regulated Kac proteins

**Table S9** KEGG pathway of down‐regulated Kac proteins

## Data Availability

The data that support the findings of this study are available from the corresponding author upon reasonable request.
