# Supplementary figures and images for: Comparative acetylomic analysis reveals differentially acetylated proteins regulating fungal metabolism in hypovirus‐infected chestnut blight fungus
Source: Mol Plant Pathol. 2023 Jun 6;24(9):1126–38. doi: 10.1111/mpp.13358 (PMC10423328; doi:10.1111/mpp.13358)

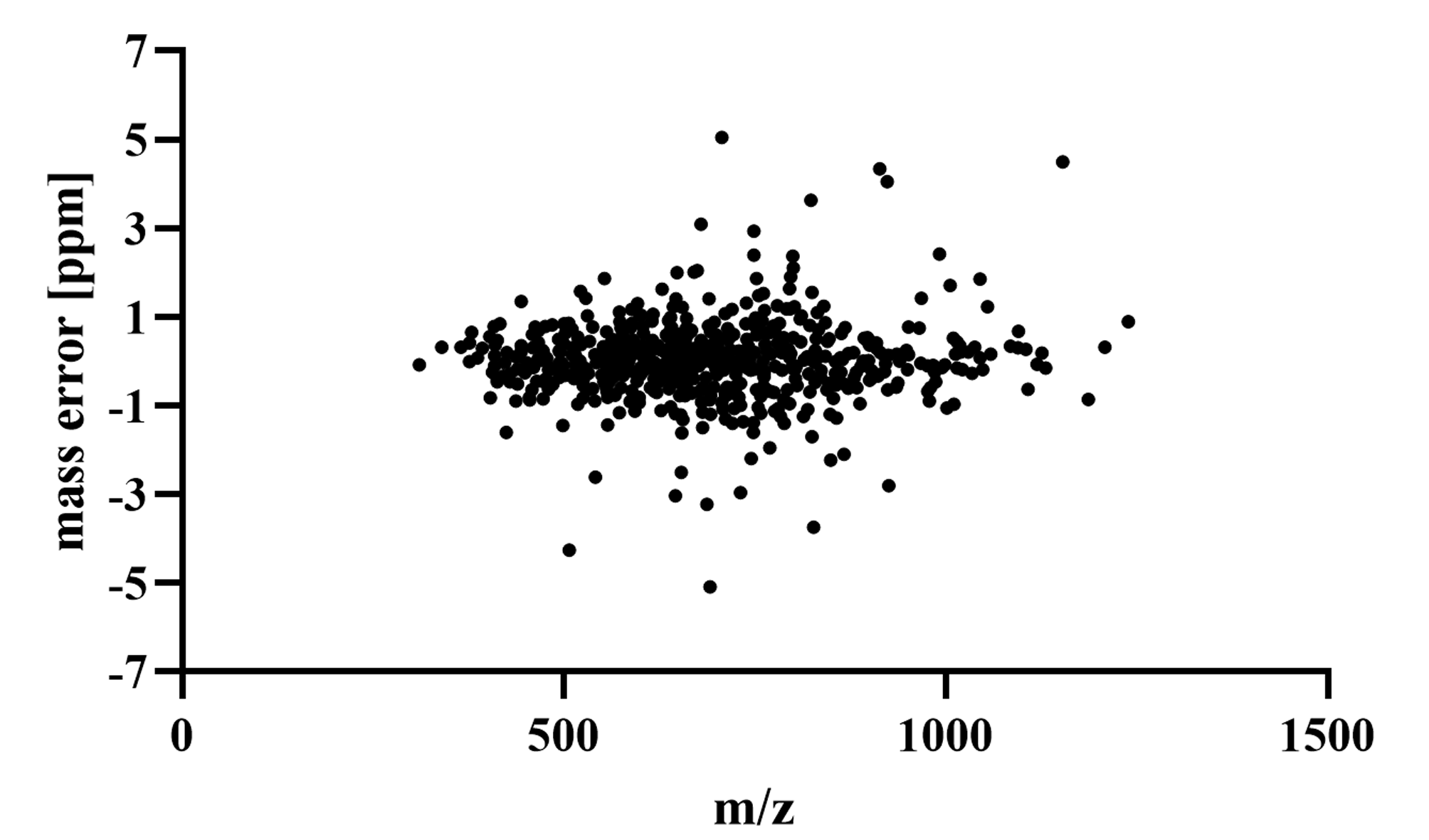

Supplement: Supplementary file 1 — Figure S1 The distribution of peptides mass error (ppm) based on m/z of 774 identified acetylation peptides [file MPP-24-1126-s002.tif]

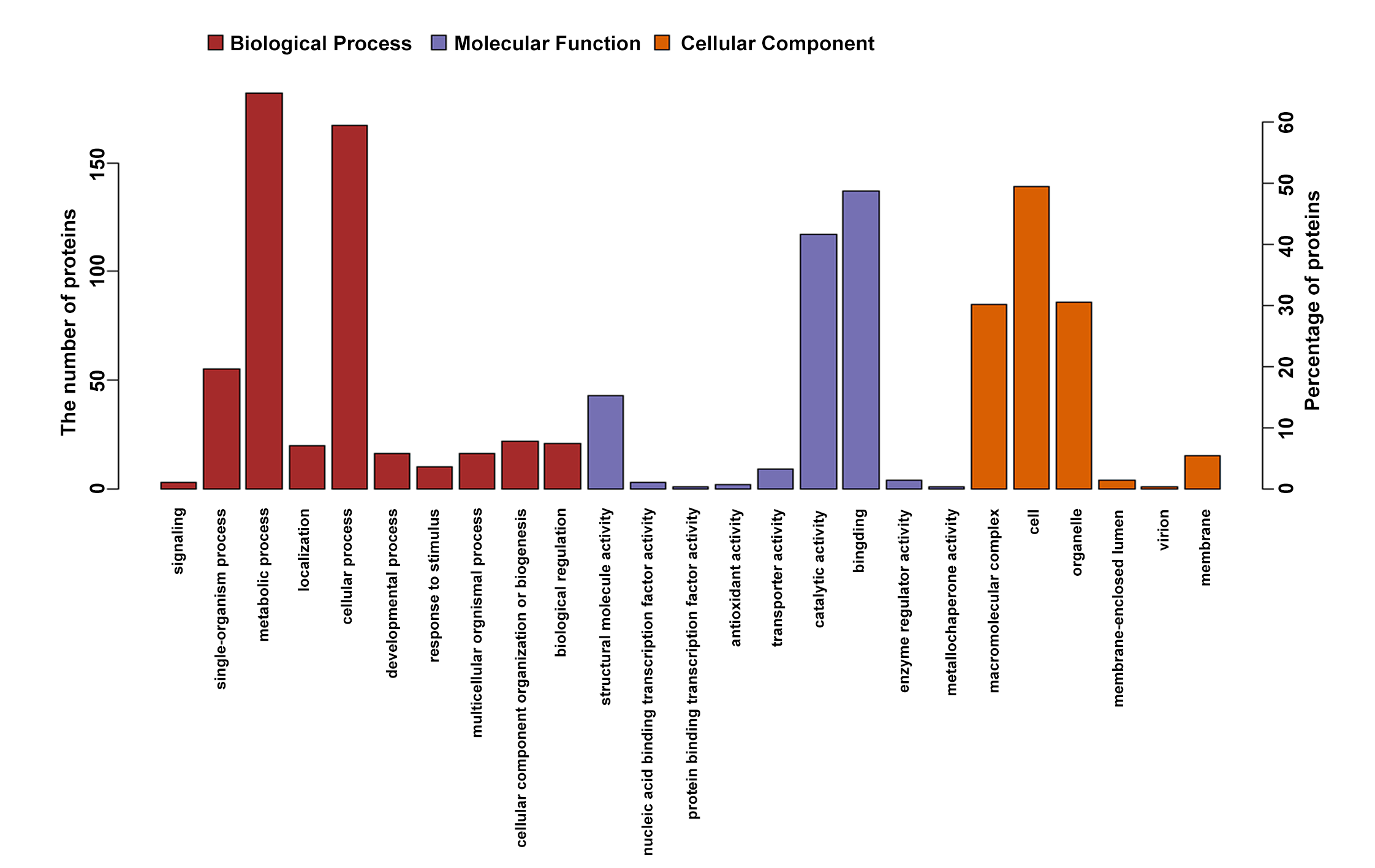

Supplement: Supplementary file 2 — Figure S2 Gene Ontology (GO) classification analysis of the differentially expressed acetylated proteins [file MPP-24-1126-s014.tif]

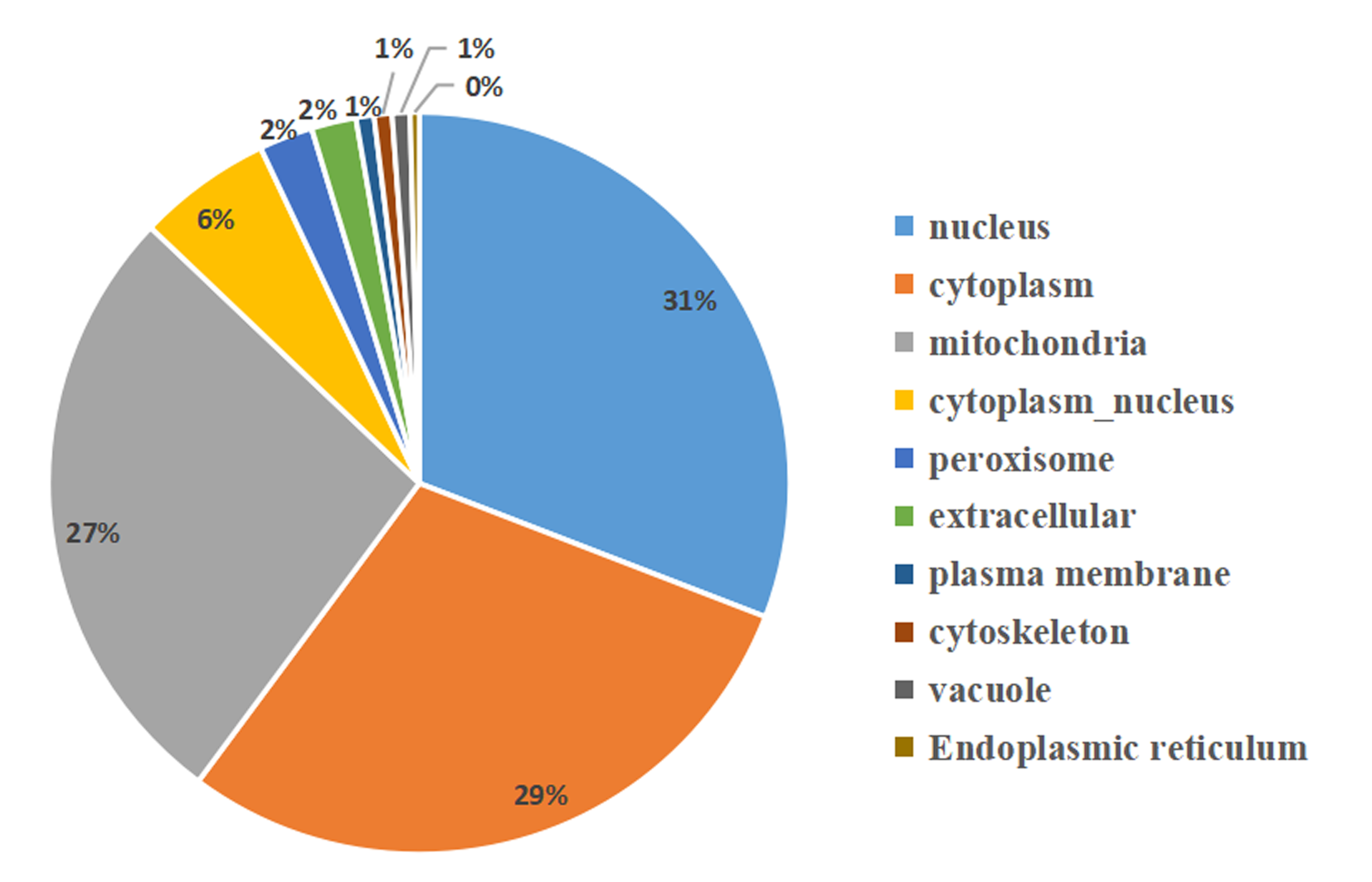

Supplement: Supplementary file 3 — Figure S3 Subcellular location prediction of the differentially expressed acetylated proteins [file MPP-24-1126-s010.tif]

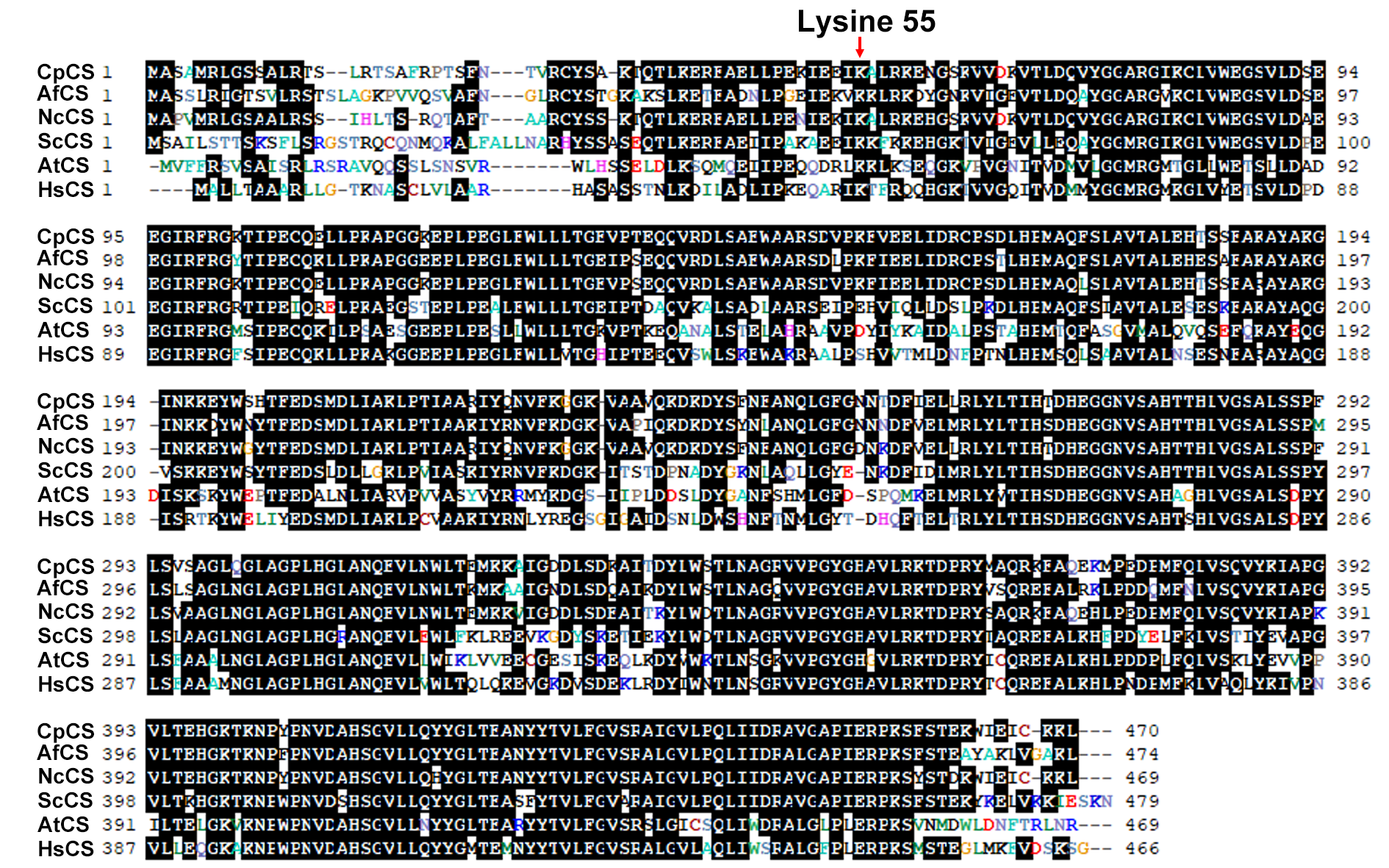

Supplement: Supplementary file 4 — Figure S4 Amino acid sequence alignment of CpCS and its orthologues: Cryphonectria parasitica CpCS (JGI 104411), Aspergillus flavus AfCS (XP_041146416.1), Neurospora crassa NcCS (XP_956898.1), Saccharomyces cerevisiae ScCit1 (NP_014398.1), Arabidopsis thaliana AtCS (NP_001327513.1), and Homo sapiens HsCS (NP_004068.2). The alignment was made using MegAlign in ClustalW. The red arrow represents lysine 55 of CpCS [file MPP-24-1126-s001.tif]

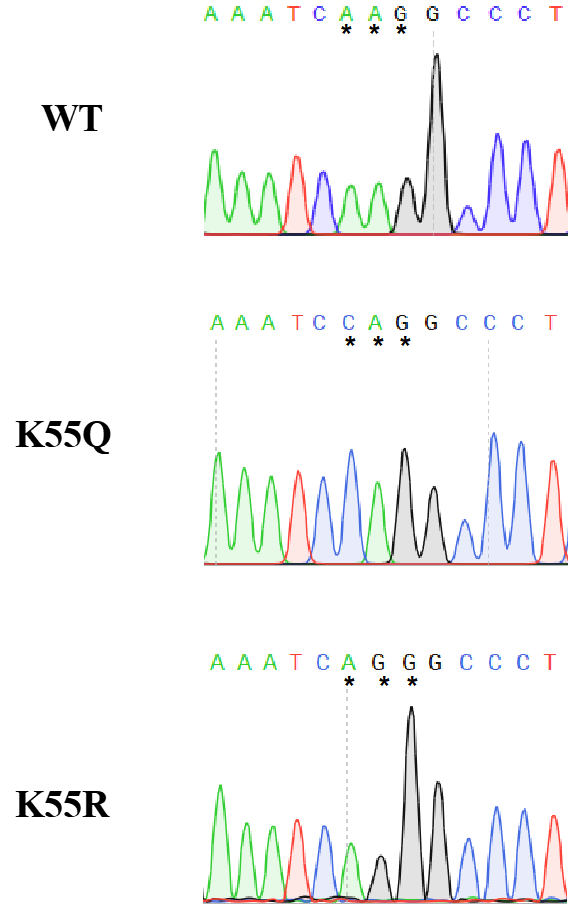

Supplement: Supplementary file 5 — Figure S5 DNA sequencing results of CpCS mutations on the pGEX‐4T‐1‐CpCS vector [file MPP-24-1126-s006.tif]

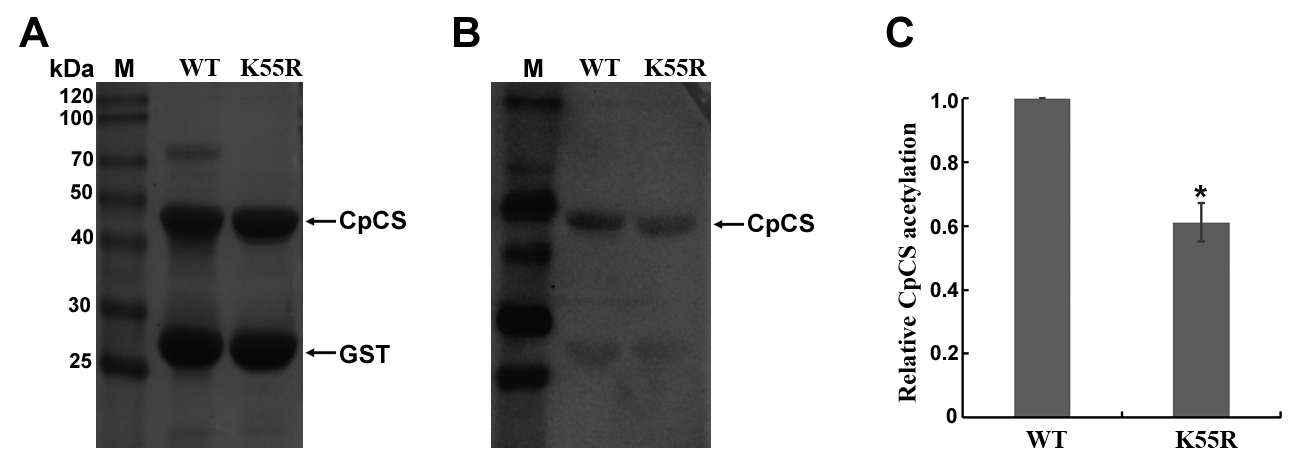

Supplement: Supplementary file 6 — Figure S6 Lysine 55 mutation reduces the acetylation of CpCS. Experiments were replicated three times and representative results are shown. (a) SDS‐PAGE of samples. (b) The acetylation levels of the two proteins were determined by western blotting using an anti‐acetyl‐lysine antibody. (c) The acetylation levels of CpCS were quantified from the western blot using ImageJ and normalized relative to the value obtained with the wild type (WT) (lane 1). The asterisk indicates a statistically significant difference from lane 1 (p ≤ 0.05, t test) [file MPP-24-1126-s007.tif]

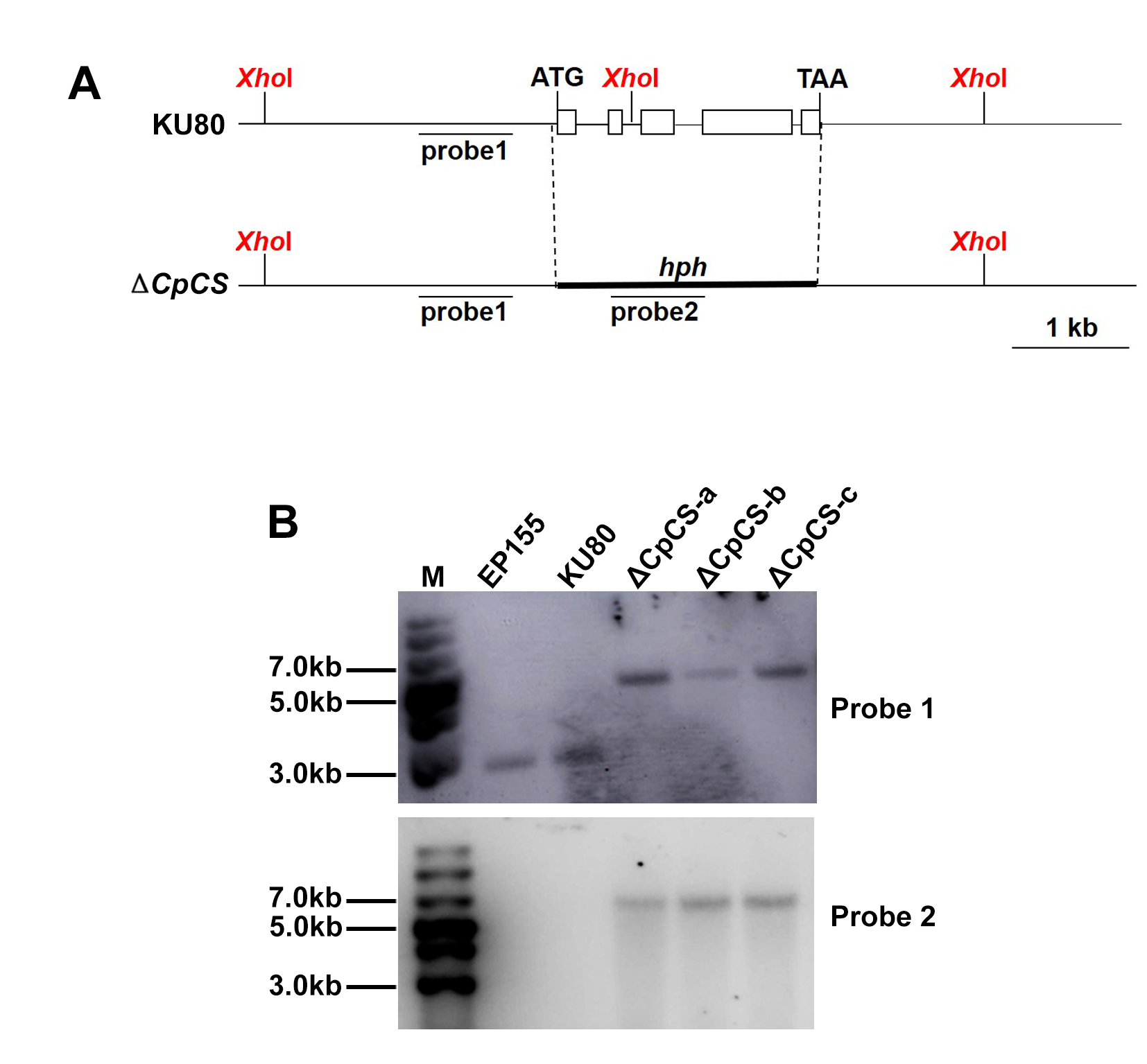

Supplement: Supplementary file 7 — Figure S7 Southern blot analysis of CpCS knockout mutants. (a) Schematic representation of CpCS gene replacement approach. Fragment on the left arm (probe 1) and fragment on the hph gene (probe 2) were used in the Southern blot analysis to distinguish the fragment size of the wild‐type strain and CpCS knockout mutants. Scale bar = 1 kb. (b) Southern blot analysis of CpCS knockout mutants. Fungal total DNAs were digested with XhoI and separated in a 1.0% agarose gel by electrophoresis, then blotted using probe 1 and probe 2, respectively. Fragment sizes are indicated in the figure margins [file MPP-24-1126-s013.tif]
